# Rapid Perceptual Switching of a Reversible Biological Figure

**DOI:** 10.1371/journal.pone.0003982

**Published:** 2008-12-18

**Authors:** Stuart Jackson, Fred Cummins, Nuala Brady

**Affiliations:** 1 Cognitive Science, UCD School of Computer Science and Informatics, University College Dublin, Belfield, Dublin, Ireland, United Kingdom; 2 Perception Lab, UCD School of Psychology, University College Dublin, Belfield, Dublin, Ireland, United Kingdom; University of Minnesota, United States of America

## Abstract

Certain visual stimuli can give rise to contradictory perceptions. In this paper we examine the temporal dynamics of perceptual reversals experienced with biological motion, comparing these dynamics to those observed with other ambiguous structure from motion (SFM) stimuli. In our first experiment, naïve observers monitored perceptual alternations with an ambiguous rotating walker, a figure that randomly alternates between walking in clockwise (CW) and counter-clockwise (CCW) directions. While the number of reported reversals varied between observers, the observed dynamics (distribution of dominance durations, CW/CCW proportions) were comparable to those experienced with an ambiguous kinetic depth cylinder. In a second experiment, we compared reversal profiles with rotating and standard point-light walkers (i.e. non-rotating). Over multiple test repetitions, three out of four observers experienced consistently shorter mean percept durations with the rotating walker, suggesting that the added rotational component may speed up reversal rates with biomotion. For both stimuli, the drift in alternation rate across trial and across repetition was minimal. In our final experiment, we investigated whether reversals with the rotating walker and a non-biological object with similar global dimensions (rotating cuboid) occur at random phases of the rotation cycle. We found evidence that some observers experience peaks in the distribution of response locations that are relatively stable across sessions. Using control data, we discuss the role of eye movements in the development of these reversal patterns, and the related role of exogenous stimulus characteristics. In summary, we have demonstrated that the temporal dynamics of reversal with biological motion are similar to other forms of ambiguous SFM. We conclude that perceptual switching with biological motion is a robust bistable phenomenon.

## Introduction

To probe the dynamics of conscious visual awareness, researchers often take advantage of bistable visual phenomena such as binocular rivalry [1], [2] and ambiguous structure from motion (SFM) [3]. By keeping input to the visual system constant, fluctuations in perception can be related directly to underlying neural processes in the brain [1]–[5]. An important first step in the investigation of any bistable stimulus is to gain a detailed understanding of the temporal dynamics of the alternation process. For example, the alternation dynamics may vary in different ways over shorter and longer timescales [6], [7], and may exhibit aspects that differ across observers [8]. The alternation dynamics also naturally vary from stimulus to stimulus; thus to gain a thorough understanding, comparison with other bistable phenomena is important [6]. In this paper we examine in detail perceptual reversal with biological motion [9], a type of structure from motion stimulus that is known to activate various high-level visual cortical regions [10]–[14]. We examine the dynamics for different types of biomotion display, and compare these to reversal dynamics experienced with other ambiguous SFM stimuli.

Biological motion stimuli, or point-light walkers, were originally developed by Johansson [9] as a means for studying observers' perception of human actions from degraded stimulus input. Researchers have since located several visual cortical regions supporting biological motion perception. Key among these are the extrastriate body area (EBA), a ventral visual region selective for human bodies [10], and posterior superior temporal sulcus (pSTS), an area selectively responsive to biological motion in humans [11], [12], as well as the various visuo-motor regions involved in action recognition [13]. Like classic experimental stimuli such as the rotating cylinder [15] or Necker Cube [16], biomotion stimuli lack the depth information given by occlusion, looming and perspective cues, and can give rise to more than one possible percept, via mirror-reversal through the image plane [17]. Yet, viewing of standard biomotion stimuli rarely gives rise to spontaneous perceptual reversal, and such stimuli have only recently been employed in studying multistable perception [17], [18].

**Figure 1:**
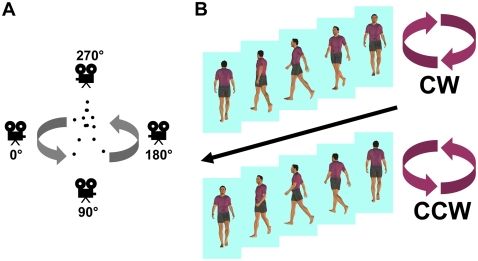
Stimulus design. (A) We created the stimulus (Movie S1) by plotting the 3-D coordinates of a standard biomotion walker, and rotating the viewing angle through one revolution during a single step-cycle. (B) As each frame of the sequence is compatible with two possible body configurations (via mirror reversal through the image plane), the walker appears to randomly reverse direction between clockwise and counter-clockwise when played over many cycles.

Here we present findings obtained with a new ambiguous biological figure developed recently in our lab, demonstrating for the first time the rapid and repeated perceptual switching that can occur with biological motion. In our first experiment, we orthographically projected a standard motion capture recording of a forward-facing human walker [19], [20] while rotating the camera viewing angle through 360° during a single step-cycle (Figure 1A; Movie S1). The resulting walker appears to rotate around a vertical central axis. Without unambiguous cues to depth, the direction of this rotating walker remains ambiguous; the changing 2-D pattern can be interpreted as a walker rotating in either clockwise (CW) or counter-clockwise (CCW) directions, with the respective interpretations having opposite starting orientations relative to the observer (Figure 1B).

## Results

### Experiment 1 Establishing the stimulus' reversible nature

To investigate the ambiguous nature of the stimulus in Experiment 1, we asked naïve observers (n = 17) to respond via button responses to perceived changes in the walker's direction over long, continuous viewing periods (600 s). Observers were not aware of the nature of the stimulus, but were instead instructed that the walker on screen would change walking direction randomly between clockwise (CW) and counter-clockwise (CCW), sometimes with longer periods between consecutive reversals and other times with shorter periods. Observers also completed analogous trials with an ambiguous rotating cylinder. Further experimental procedures are detailed in Materials and Methods.

**Figure 2:**
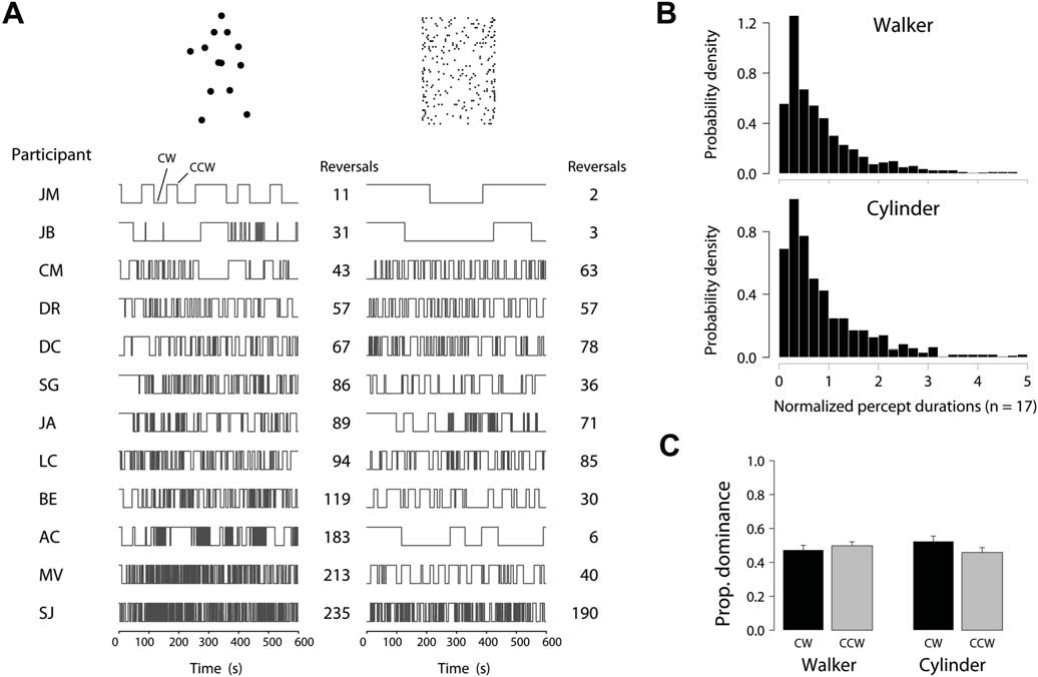
Experiment 1 results. (A) Alternation of the stimuli between clockwise and counter-clockwise over 600 s trials. Participants experienced quite varied numbers of reversals for both stimuli. Several participants reported transient phases in which the walker briefly ‘collapsed’ or ‘danced’ (see Materials and Methods); for 3 of the 17 participants these periods accounted for approximately 10%–20% of trial time. For the other 14 participants, these periods were rarely experienced and accounted for less than 1% of total trial time. (B) Normalized percept durations across all observers is plotted (each observer's durations divided by the mean duration for that participant). For both stimuli we see a distribution with a particularly fast rise and rightward tail. Durations in which neither CW nor CCW were perceived were excluded from this analysis. By multiplying bin height by bin width (0.2 units each), we get the proportion of all responses falling in a particular bin. The horizontal axis upper limit is set at 5 normalized units for illustration; a very small number of longer normalized durations are not shown for this reason. (C) Mean proportion dominance time for CW/CCW percepts for the walker and cylinder stimuli (n = 17, 170 mins total per stimulus). Error bars indicate standard error.

The results of this first experiment are illustrated in Figure 2. Continuous viewing of the rotating walker led to significant levels of conscious perceptual switching in most observers tested, sometimes several hundred reversals occurring over the 600 s trials (Figure 2A). Perceived reversal rate varied by an order of magnitude across observers, a measure common to other multistable visual phenomena [5]. Most participants were quite surprised when told afterwards that the walker never actually changed direction; changes were generally reported as being quite perceptually convincing. Pairing the walker against the rotating cylinder (Figures 2A–2C), we found comparable levels of percept-reversal for both figures in several observers, although this relation did not hold for everyone tested (r = 0.515, p<0.05). Overall, reversal rates with the cylinder were lower (mean reversals/min±s.e. - Walker: 8.4±1.6; Cylinder: 4.6±1.1). Note that cylinder rotation speed (4 s period) was kept several times slower than the walker's 1 s cycle period, as pilot studies indicated cylinder periods of 1 s–2 s were difficult to monitor due to the much larger array of stimulus dots. Examining the distribution of normalized percept durations (Figure 2B) and dominance times for CW/CCW percepts (Figure 2C), we found comparable profiles for both figures.

Table 1 presents the number of reversals and first percept durations experienced by each of the 17 observers. Despite dominance times for CW/CCW being approximately equal for both stimuli (Figure 2C), an asymmetry is apparent in the distribution of first percepts. Fifteen out of seventeen observers' first percept with the walker was of CCW rotation; that is, of the walker facing directly away on initial appearance, and the body axis gradually rotating around to face the observer over the subsequent frames (see Figure 1B, lower sequence; slowly cycle through the frames of Movie S1). This trend is highly significant (p<0.005, binomial test). With the kinetic depth cylinder, in contrast, eight out of seventeen observers' first percept was of CCW rotation, the other nine perceiving CW at onset. Strong onset effects are known to occur for other bistable stimuli, such as observers' bias for the coherent percept at the onset of plaid stimuli [21]. With the walker, we believe the onset bias may be related to the figure's orientation on first appearance, and in unpublished work we have noted that modifying onset orientation may alter the likelihood of perceiving CW or CCW at stimulus onset.

**Table 1:** Summary of reversal rates and first percepts in Experiment 1.

Observer ID	Walker		Cylinder	
	No. of reversals (mean duration)	First percept (duration)	No. of reversals (mean duration)	First percept (duration)
SJ	235 (2.5)	CCW (3.5)	190 (3.1)	CW (12)
MV	213 (2.8)	CCW (9.5)	40 (14.3)	CW (12.7)
AC	183 (3.2)	CCW (9.4)	6 (98.4)	CCW (116.1)
BE	119 (5)	CCW (14.4)	30 (19.2)	CW (22.9)
LC	94 (5.2)	CCW (4.9)	85 (6.5)	CW (12.1)
JA	89 (6.7)	CCW (47.6)	71 (8)	CCW (98.3)
SG	86 (6.8)	CCW (67.1)	36 (15.6)	CCW (12)
DC	67 (8.9)	CW (17.6)	78 (7.6)	CW (7.4)
ED	67 (8.8)	CCW (7.8)	63 (8.8)	CCW (4.8)
RR	60 (8.3)	CCW (27.4)	5 (96.2)	CCW (20)
DR	57 (10.2)	CCW (19.1)	57 (10.3)	CW (8.6)
JD	44 (11.7)	CW (41.2)	4 (136.3)	CW (266.6)
CM	43 (12.9)	CCW (5.3)	63 (9.5)	CW (26.7)
JB	31 (19.2)	CCW (48)	3 (150.9)	CCW (126.8)
EB	26 (22.4)	CCW (62.3)	30 (19.9)	CW (222.4)
JM	11 (49.5)	CCW (8.1)	2 (193.9)	CCW (210.7)
BR	9 (61.5)	CCW (93.6)	24 (24.7)	CCW (24.7)

‘Mean duration x No. of reversals’ is less than 600 s, as the final percept was artificially ended and is therefore not included in analyses. For lower switch trials, this accounts for 30 s or more in many cases. In addition, durations in which neither CW nor CCW were perceived are not included. Mean durations are given in seconds, rounded to one decimal place.

### Experiment 2 Biological and Rotational Motions

A detailed understanding of the temporal dynamics of perceptual bistability requires a sufficiently large distribution of perceptual reversals, and knowledge of how learning affects these distributions over time [6]. Recent findings suggest that, for many bistable phenomena, observers experience consistent patterns of reversal rate drift (i.e. increase or decrease in reversal rate) within and across viewing sessions. Van Ee [6] recently found that for perceptual rivalry (slant rivalry, Necker cube reversal), as well as two different types of binocular rivalry (orthogonal grating, house-face rivalry), the alternation dynamics show reasonably reliable patterns of drift. Across test repetitions, for example, the drift was not significantly different from zero for any of the four stimuli. The drift across successive 35 s portions of the data, on the other hand, showed a small but consistent negative slope for each stimulus, indicating that reversal rates typically decrease over a trial. Similar results were recently obtained by Suzuki & Grabowecky [7], using simple ‘×’ and ‘+’ shapes as rivaling stimuli. For example, reversal rate tended to decrease over short 20 s exposures to the stimuli, while over a full session of 20 s trials, reversal rates were stable. Across multiple sessions (separated by an average of 1.7 days), in contrast, reversal rates generally increased.

How do the temporal dynamics of reversals with the rotating walker vary over different time periods, and how do these dynamics compare with the bistability intrinsic to a standard point-light walker (i.e. non-rotating)? By adding a rotational component to a biomotion walker, the stimulus now contains two main motion components: the local joint motions i.e. regular ‘biomotion’, and the somewhat artificial rotation. How do these components interact to produce the significant degree of bistability observed with the rotating stimulus in Experiment 1? One possibility is that the additional image motion created by the relatively fast rotation (360°/s), will lead to a general ‘speeding’ of reversal rate. Reversals with other SFM stimuli are known to vary with angular velocity of the display [22]. In that study, the authors systematically varied the angular velocity of a rotating globe, with velocities ranging between 16–80°/s. The authors found that perceptual durations generally decreased as a function of increased angular velocity, with the phase distribution peaks shifted to shorter mean durations at faster angular velocities. In addition, the speeding of reversal operated across all individual percept durations, lessening longer and shorter durations in proportion.

To investigate the stability in reversal rates over time, and to compare dynamics for rotating and standard biological motion, we tested a group of observers on trials with standard walker and rotating walker stimuli. For standard biomotion, we presented a walker oriented constantly at 30° from frontal orientation, as though facing over the observer's left shoulder (see links in [17] for a demonstration of the intrinsic bistability in standard biomotion stimuli). Observers responded to reversals from the frontwards-facing percept (30° left) to the backwards-facing percept (150° left), and vice-versa, and were given sufficient practice with the stimuli so that they had no difficulty experiencing reversal. This was particularly important for the standard walker, as many observers find it difficult to see the backwards-facing percept on their first attempt. Four observers, including one author, participated in the experiment. A session consisted of sixteen 120 s trials, with eight trials for each type of stimulus. To investigate the consistency in the data over time, each observer completed four repetitions of the experiment, with no more than two repetitions per day (mean gap between repetitions was 1.9 days). Control trials were also completed in the first experimental repetition (see Materials & Methods). In total we collected 512 minutes of data in this experiment (4 observers×4 repetitions×16 trials×2 mins), giving a total of 7,451 reversals from these four observers.

**Figure 3:**
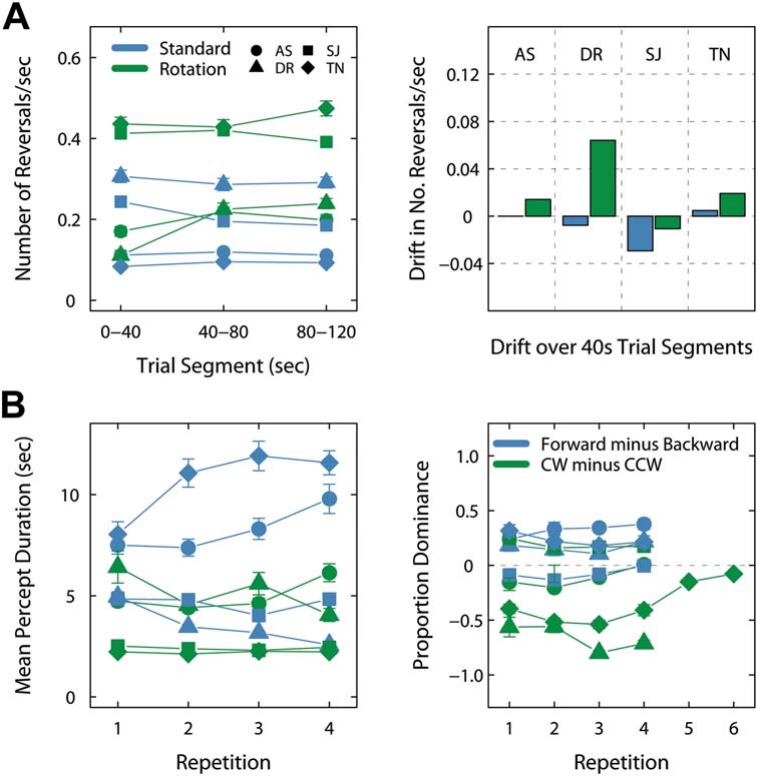
Experiment 2 results. (A) Reversal rates per second (left) and drift (right) across the 120 s trials, broken down into successive 40 s segments. Drift is given as the mean increase or decrease in revs/s between segments 1–2, and segments 2–3. Individual observers are represented by different symbols. Data for the standard walker (blue) and rotating walker (green) are presented separately. (B) Observers completed four repetitions of the experiment. The mean percept durations for each session (8 trials per stimulus) are plotted on the left. Proportion dominance for the different percepts across all sessions is given on the right. Positive values indicate greater dominance of the CW (rotating) or frontwards-facing (standard) percepts. We tested observer TN in an additional two sessions with the rotating walker, during which greater retention of the less dominant percept was encouraged. This resulted in a more even distribution of dominance proportions. Error bars indicate standard error throughout.

When we segment the trials into successive 40 s periods, reversal rates for both stimuli vary in quite a stable fashion for individual observers (Figure 3A). The drift (in reversals/sec) is relatively small for most stimulus/observer pairings. For three of the four observers there is a minimal negative slope or no slope in reversal with the standard walker. For the rotating stimulus, two observers experience a slight increase in reversal over the trials, with another observer experiencing a much larger mean increase. We note that this observer's onset percept durations with the rotating walker were significantly longer than any other observer's, which may underlie this large positive drift. The drift in reversal rate is generally small across test repetitions also (Figure 3B). Three observers experienced consistently longer mean percept durations with the standard walker. This result suggests that the added rotational motion leads to an increase in reversal rates with biomotion. However, this speeding does not necessarily operate equally across all durations (i.e. making all percept durations shorter), as the differences in within-trial drift indicate. However, while we collected quite extensive data per individual, the relatively small number of observers studied here (n = 4) may limit the strength of conclusions that can be drawn.

**Figure 4:**
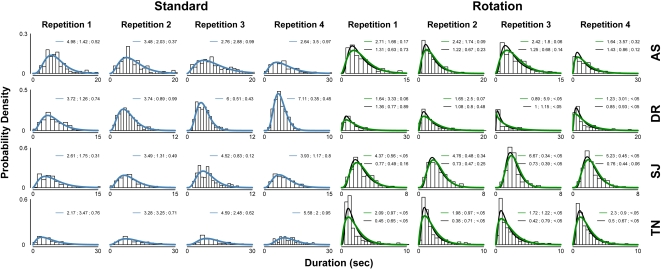
Experiment 2 phase distributions. Gamma distribution fits (blue) for the standard walker data are presented on the left-hand side, and both gamma (green) and lognormal (black) fits for the rotating walker appear on the right. In applying these fits, some data points at the upper extreme (2% longest durations) were excluded to improve the quality of the fit [33]; however, the 2% shortest durations were retained, as quite often observers' experienced percept durations less than 1 s, making the filtering of error button responses problematic. The distributions contain on average over 200 data points. The numbers above each histogram represent the fitted parameters and quality of fit: shape, scale, p-value (gamma); mu, sigma, p-value (lognormal). A p-value greater than 0.05 given by the Kolmogorov-Smirnov test represents a fit of acceptable quality.

Figure 4 presents the gamma distribution fits for each observer's data. In all cases, the fit was satisfactory for the standard walker data, as given by a Kolmogorov-Smirnov goodness-of-fit test. However, the gamma fit for the rotating walker data was satisfactory in only seven out of sixteen cases; a lognormal distribution provided a better fit in several of these (but still not all). The maximums for these distributions rise extremely above the surrounding bins. Again, we see that the difference between alternations with the standard and rotating stimuli may be qualitative as well as quantitative. Either way, the consistent patterns in these data within and across repetitions are reminiscent of results with other bistable stimuli [6], [7], proving that biological motion can provide a reliable stimulus for studies on bistable perception.

### Experiment 3 Object-Type and Reversal Location

In early work on the kinetic depth effect, Wallach & O'Connell [15] studied observers' perception of the shadows created by rotating solids and wire figures. In their first experiment, observers were shown the shadows of a rotating block “in the shape of a roof with sloping gables” [p. 207, see their Figure 1]. The authors note that “occasionally an S [subject] sees for long periods reversals after each rotation of 180°” [p. 207]. Although the authors do not specify this, we believe they may have been remarking on the propensity for reversals with non-uniform, deforming structures, to occur at certain phases of the figure's rotation cycle more than others (i.e. to be related in some way to the figure's geometry). This might lead to perception becoming trapped between one or more reversal hotspots, separated by a fixed angle of rotation e.g. by 180° in the case of the sloping gables. In studying the role of endogenous and exogenous factors on rotating globe reversal, Brouwer & van Ee [22] recently made the interesting observation that the inclusion of local patches of high density (or gap-patches i.e. no dots) triggers alternations towards the direction of the moving patch. Additionally, the authors found some evidence that the patches triggered reversals more frequently when the patch was at a particular angular location, although this location differed for different observers. The authors noted how this resulted in perceptual phases tending “to last one, or multiples of one, full rotation” [p. 3398].

Over the course of our investigations, many observers have reported periods in which the rotating walker “toggles” rapidly between CW and CCW, reversing direction after roughly consistent periods of rotational motion (e.g. after each rotation of 360°; this percept is distinct from the collapsing percept that some observers perceive, but is sometimes reported to precede it). We hypothesise that this perceptual toggling with the walker may be linked with particular phases of the rotation cycle, and may be similar in nature to the phenomenon hinted at by Wallach & O'Connell [15]. The phenomenon may or may not be similar in nature to the findings that emerged from Brouwer & van Ee's [22] study on reversal with a modified rotating globe, in that the non-uniformity of the biomotion walker (i.e. the fact that it is not a uniformly dense surface of dots) may give rise to a non-uniform distribution of responses over the rotation cycle. To investigate this, we initially re-analysed the data from our first two experiments. We then carried out an experiment comparing reversal location distributions for the rotating walker and a non-biological object with similar global dimensions (‘rotating cuboid’). This allowed us to investigate whether perceptual toggling occurs more generally for reversible figures that produce non-uniform image projections during rotation. This issue is important as it may be another instance of exogenous stimulus characteristics having a role in bistable vision [22]. However, this does not rule out the possibility that these exogenous effects operate by endogenous means (e.g. via eye movements), an issue which we will also discuss.

**Figure 5:**
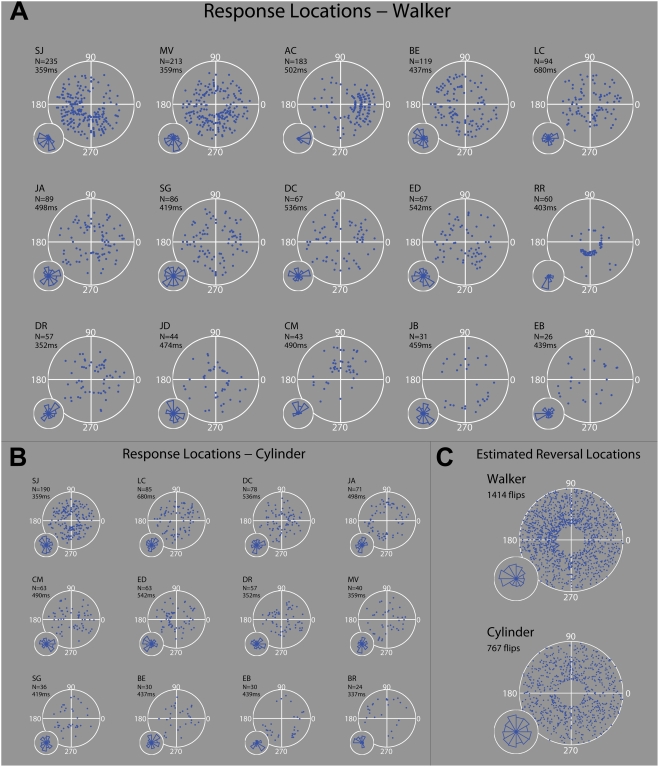
Response location distributions. (A) The polar plots depict the locations of observers' button responses relative to the walker's rotation cycle (Experiment 1). Distance from the centre of the polar plot illustrates how far in time into a trial a response occurred i.e. successive rotation periods span out from the centre in concentric pattern. Note that an artificial gap is added to the centre of each plot, to avoid crowding of dots in the centre. Histograms (inset; 30° bins) illustrate the proportion of these responses falling in set angular segments of the rotation. The plots are given in order of reversal frequency (high frequency trials at top). For space constraints, data from some very low switchers (approx. 1 rev/min or less over the ten-minute trials) are not included. An estimate of each observer's reaction time, taken from the mean RT in a control trial, is given above each plot. (B) Reversal location distributions for the cylinder trials (extremely low switchers excluded). (C) From each observer's data, we estimated the walker's orientation at the time of perceptual reversal by subtracting a set angular amount from the response location data, based on the observer's mean reaction time in a control trial (1 rotation cycle = 1000 ms). The plots illustrate the RT-corrected data pooled from all observers. Data for the cylinder trials is also presented; however, as each observer viewed a randomly generated cylinder (with ‘wraparound’ of dots at either edge), a strict comparison between the two stimuli may not be appropriate. The cylinder data may best be considered an example of a randomly distributed dataset.

Figures 5–7 present analyses of the data from our first two experiments. For the single trial data from Experiment 1 (Figure 5), polar plots depict the locations of observers' button responses relative to the stimulus' rotation cycle (i.e. the phase of the rotation cycle at the time of button response). The smaller polar histograms (inset) illustrate the proportion of these button responses made within set angular regions (30° bins) of the cycle. The plots are given in order of reversal frequency (high frequency trials at top). Due to space constraints, data from the very lowest switchers (approx. 1 rev/min or less over the ten-minute trials) are not included. An estimate of each observer's reaction time, taken from the mean RT in a control trial, is given above each plot. From studying the plots, it is clear that for some of the high switchers in Experiment 1, but not all, responses are not evenly distributed across the 60-frame rotation cycle. Consider the similarity in the distribution of walker responses for the naive observer MV and author SJ, given in the upper row. Each experienced more than two hundred reversals, with button responses congregating in or near the third quadrant of the polar plot (180°–270°). Note also the data of RR on the second row, in which a very large proportion of reversals occur in a similar region. Another significant peak occurs in the distribution of reversals for observer AC, with the vast majority of responses occurring in a segment of the walker's rotation cycle stretching approximately 60°, although in a different location to the previously mentioned observers. The high switch trials with the cylinder seem to have less defined peaks in the distribution of responses (Figure 5B).

**Figure 6 pone-0003982-g006:**
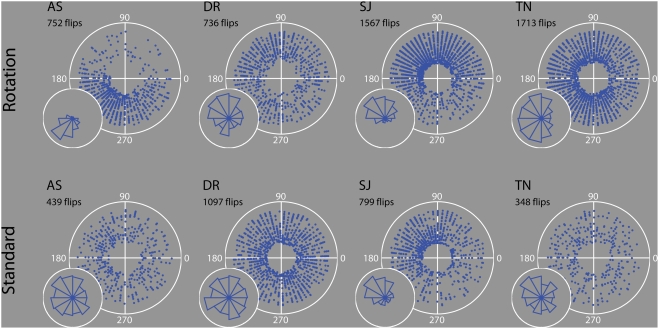
Response location distributions. Polar plots illustrate the estimated reversal locations (i.e. RT-corrected) for the rotating (upper row) and standard walker (lower row) stimuli presented in Experiment 2, combined across all repetitions. For the standard walker, angular location refers to the phase of the walker's step-cycle, which was identical to the rotating walker step-cycle. Distance from the centre of the polar plot illustrates how far in time into a trial a response occurred i.e. successive rotation periods span out from the centre in concentric pattern. Note that an artificial gap is added to the centre of each plot, to avoid crowding of dots in the centre. Chi-squared tests verified that each of the rotating walker histograms deviated significantly from a uniform distribution. Rotating–AS: χ^2^ = 644.6, p<10^−15^; DR: χ^2^ = 65.5, p<10^−9^; SJ: χ^2^ = 637.2, p<10^−15^; TN: χ^2^ = 153.1, p<10^−15^. Standard–AS: χ^2^ = 14.5, p = 0.208; DR: χ^2^ = 16.5, p = 0.12; SJ: χ^2^ = 245.5, p<10^−15^; TN: χ^2^ = 18.1, p = 0.07 (df = 11 for all).

**Figure 7:**
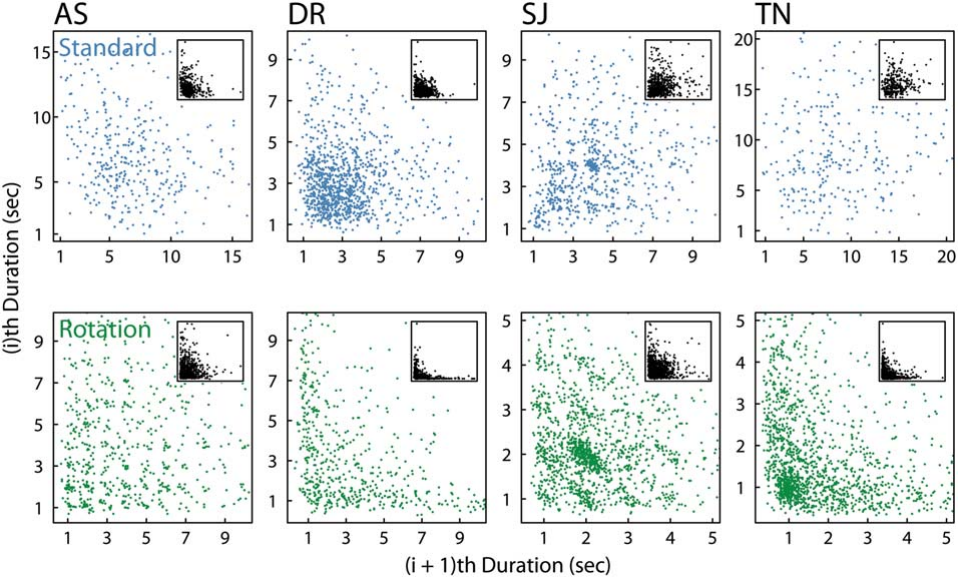
Lag plots. A simple method for checking randomness in percept duration data is the lag plot i.e. plotting the i^th^ percept duration against the (i+1)^th^ duration. The plots present this data for each observer from Experiment 2 (combined across repetitions). The inset illustrates the entire duration distributions, with the main plots depicting the kernel of these distributions.

The data combined across observers (Figure 5C), and corrected for reaction time, is more interesting. By taking into account an estimated RT for each observer, we obtain a rough estimate of the locations at which perceptual reversals occurred. Recent studies have used RT estimates very effectively in estimating the timing of percept reversal relative to eye movements [23], [24]. This analysis also resembles in spirit the reverse-correlation procedures used to study rotating globe reversal [22]. The polar plot reveals a clear asymmetry in the estimated reversal locations for the walker, with a dense band stretching from 60°–210°. A chi-squared test confirmed that the distribution of walker response data differed significantly from a uniform distribution (χ^2^ = 134.8, df = 11, p<10^−15^), while the cylinder response data did not (χ^2^ = 9.43, df = 11, p = 0.582). While these data are clearly dominated by the high-switch trials (recall that most observers were naïve in this experiment), and by no means pinpoint a reversal ‘hotspot’, they do suggest that further investigation of the issue could be revealing.

The data from Experiment 2 (Figure 6) are more extensive, and allow us to speculate on this issue more thoroughly. For each of the four observers, we present the RT-corrected plots combined across the four experimental repetitions, for the rotating and standard walker data. The standard walker plots depict responses relative to the phase of the step-cycle, which was identical to the rotating walker's step-cycle (i.e. identical except for the rotation). It is apparent that for at least three observers the responses occur more often in particular quadrants of the rotation cycle (see Figure 6 text for chi-squared test results). The RT-corrected plots suggest that the peak reversal locations may be similar for DR and SJ (2^nd^ quadrant, 90°–180°), but slightly later for observer AS (3^rd^ quadrant, 180°–270°). Each of these datasets is quite significant in size, with the largest bins containing several hundred reversals. For SJ, a similar asymmetry emerged in both the rotating and standard walker responses, indicating that the walker's periodic step-cycle (and not just the periodic rotational motion) may have a role to play. For both stimuli, it may be the case that specific movements of the walker, such as the transition from a backward-swinging to a forward-swinging limb for example, increase the salience of a particular structure/depth interpretation. It is possible that eye movements then trigger the reversal. The results from the other three observers, however, showed no obvious peaks with the standard walker.

Some debate exists about the correct use of autocorrelation analyses with percept duration data, given that durations are typically not-normally distributed [6]. We analysed the data from Experiment 2 using both the standard autocorrelation across trials (Pearson) and the Spearman rank-method across trial segments, and found quite high correlations at the first lag in all data (typically >0.25), with strong correlations continuing in the rotating walker data for several additional lags. However, we were not convinced that our autocorrelation analysis was appropriate, particularly when individual 40 s trial segments contained only a small number of percept durations, not much greater than the number of lags studied. A simple method for checking randomness in the data that avoids these issues is the lag plot i.e. plotting the i^th^ percept duration against the (i+1)^th^ duration. Figure 7 presents lag plot analyses of the data, plotting individual durations against succeeding durations. In most cases, successive durations with the standard walker appear not to be related, having a random distribution similar to the data given by other forms of bistable display (see [6] for a sample lag plot analysis with slant rivalry). For the rotating walker, in contrast, the distribution of data points is qualitatively different for all observers. First of all, note the strong patterns that emerge for the two observers who displayed a bias towards CCW rotation (DR, TN; see Figure 3B). These observers tended to experience patterns of long CCW durations interspersed with short CW rotations, leading to a lag plot distribution that follows closely the horizontal and vertical axes. For DR, the short durations are tightly packed around 1 s duration.

For observers AS and SJ, the trend towards durations of 1 s, or multiples of 1 s, is really quite apparent. Recall Brouwer & van Ee's [22] observation that perceptual phases with the high-density patch (or gap-patch) globe tended to last one, or multiples of one, full rotation [p. 3398]. Here, for both observers we see localized peaks within the distribution that fall consistently along a whole no. of seconds. For example, a percept duration of 3 s appears to be more probable than 2.5 s or 3.5 s for observer SJ, and similar trends are clear around durations of 1 s, 2 s and 4 s. For AS, the trend towards percept durations of one, or multiples of one rotation, is clear up to durations of at least 10 seconds. Histograms of the data (with 0.1 s bins) confirmed this observation. This effect is presumably related to the stimulus having a revolution period of 1 s, and our suggestion based on the polar plot analyses, that reversals might be triggered more often at particular figure orientations. Of course, the expression of these patterns may involve endogenous inputs such as eye movements, which must inevitably be triggered by the stimulus motion.

To confirm that similar distribution asymmetries occur with a non-biological stimulus, we ran three observers on trials with the rotating walker and an ambiguous rotating cuboid. The cuboid was chosen as a comparison non-biological stimulus as its overall dimensions are easily controlled (to be roughly similar to the walker's dimensions, height:width:depth ratio = 4∶2∶1). The cuboid may also be representative of the class of geometric solids studied in early research on kinetic depth [15]. Observers completed three sessions of trials; each session consisting of four blocks of six 60 s trials. Individual transition types (Switch to CW/Switch to CCW) were monitored in separate blocks, as we felt this might increase the accuracy of our reaction time estimates in pinpointing reversal locations (by reducing the crowding of consecutive button responses on each other). Further details are given in Materials and Methods.

**Figure 8:**
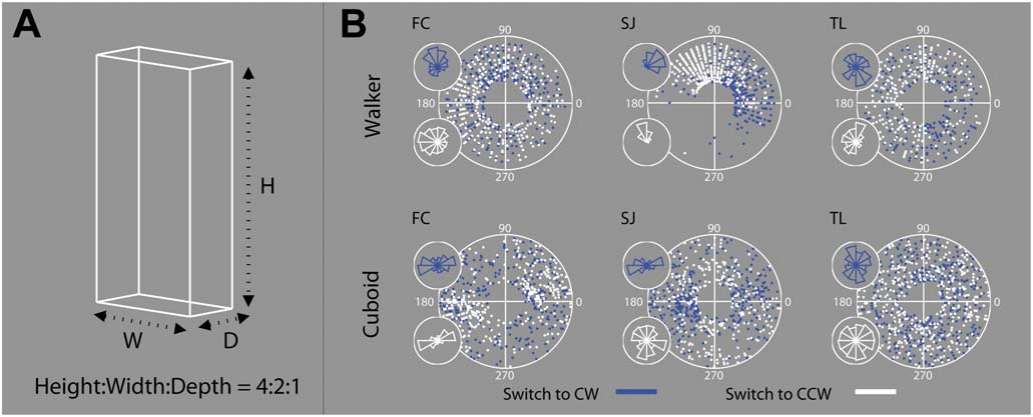
Object-type and reversal location. (A) An ambiguous rotating cuboid was presented in half of the trials in Experiment 3. The cuboid was presented as though the observer was looking down on the top of the object from a 10° elevation. Without this a 3-D structure is not visible under rotation (instead only the four vertices are seen, translating as lines). A side effect of this elevation is that the percept of ‘looking down’ on the cuboid is generally more prevalent than the alternative percept [34]. As our focus in Experiment 3 was on the locations of percept transition, and not dominance durations per se, we ignored this aspect of the cuboid's reversal dynamics. (B) Switch to CW (dark blue dots) and Switch to CCW (white dots) were monitored in separate blocks of six 1-min trials. Four blocks were completed in each repetition, one for each transition-stimulus pairing, presented randomly.

Figure 8 presents the results of this experiment. For observer FC, responses clearly occurred in higher density at particular phases of the cuboid's cycle (Switch to CW: χ^2^ = 62, df = 11, p<10^−8^; Switch to CCW: χ^2^ = 210.5, df = 11, p<10^−15^). However, in contrast to the single-peaked distributions generally seen with the walker, two reversal location peaks emerged, with roughly 180° of separation. These peaks were relatively stable across the three sessions. A similar distribution is apparent for SJ, but this time most significantly for the Switch to CW transitions (Switch to CW: χ^2^ = 186.1, df = 11, p<10^−15^; Switch to CCW: χ^2^ = 23.1, df = 11, p<0.05). Finally, for TL strong distribution asymmetries are not as apparent in the cuboid data, although the distribution of transitions to CW differed significantly from a uniform distribution (Switch to CW: χ^2^ = 22.2, df = 11, p<0.05; Switch to CCW: χ^2^ = 5.83, df = 11, p = 0.88). These results confirm that peaks in response location distributions can occur with other rotating geometric forms such as the cuboid. However, as with the walker, these reversal patterns may be observer (and possibly transition) specific.

### Rotation Speed and Eye Movements

So far we have only documented the existence of reversal location peaks; we have not attempted to explain exactly how these peaks come about. Two probable factors here are (i) asymmetries in the distribution of image motion across the rotation cycle (due to stimulus non-uniformities) and (ii) the related issue of eye movement patterns.

**Figure 9:**
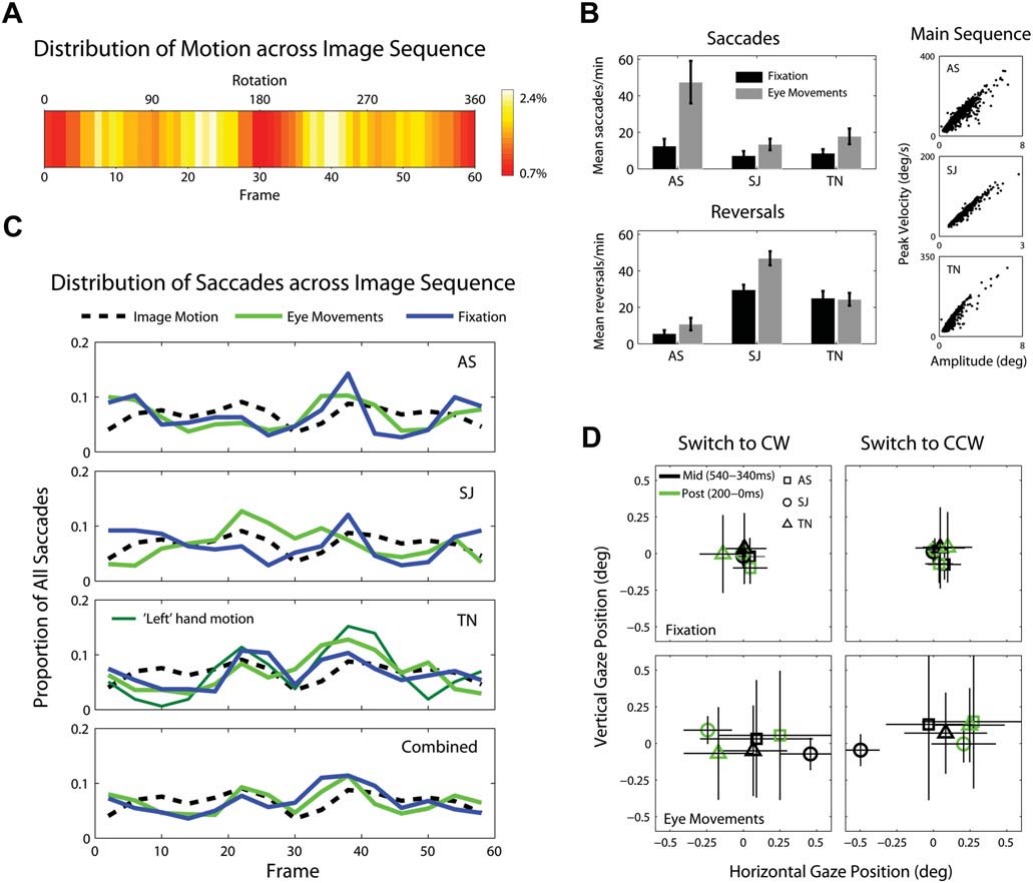
Eye movements and rotation speed. (A) For each individual dot making up the walker, we calculated the absolute horizontal distance traveled (in pixels) from frame to frame i.e. leftward and rightward motion are treated similarly. Combining the values for all thirteen dots at each frame, and dividing by the total motion in pixels over all frames, gives the proportion of image motion contained between any two frames in the rotation cycle. The colormap illustrates the distribution of image motion across the 60-frame sequence, with brighter values (white) indicating a greater proportion of image motion. (B) Mean saccades/min and reversals/min for each of the three observers, in the fixation (black) and eye movements encouraged (grey) conditions. Error bars represent standard deviations. To confirm that our saccade detection routines performed satisfactorily, we plotted saccade peak velocity against amplitude (i.e. the main sequence [32]). (C) For all saccades made in a particular condition, we calculated the proportion triggered during the presentation of individual frames of the rotation cycle, by noting the frame on screen during the starting sample of the saccade. The blue (fixation) and green (eye movements) lines illustrate this distribution, divided into fifteen equally-sized bins. The black line represents the distribution of image motion across the sequence. Note that the combined histogram data (row four) is not the average of individual datasets, but the pooled saccade data. (D) For each percept reversal, we compared the median fixation positions during two separate time windows prior to the button response: Mid-reversal (540ms-340ms prior to response), during which the reversal is estimated to have occurred, and Post-reversal (200ms-0ms prior to response). Each symbol corresponds to the average over all reversals for a particular observer. Horizontal and vertical lines represent the inter-quartile range along the respective axes. Note than unlike in Experiment 3, both transition types were monitored as normal in each trial. The plots separate the data for each transition type for comparison.

Figure 9A depicts the distribution of walker dot motion vectors (horizontal component), normalized across the rotation cycle. We can see two obvious peaks in the distribution of absolute dot motions, at approximately one-third and two-thirds of the way through the rotation cycle (slowly cycle through Movie S1). Therefore, it is very likely that image motion may in some way influence the percept in systematic ways. Beintema, Oleksiak & van Wezel [25] have shown that interpretations of biological motion are strongly affected by the stimulus speed. These authors found that when presented at unnaturally slow speeds, biological motion is perceived to rotate in depth. This effect was particularly strong when the figure was rendered unfamiliar to observers, by scrambling or inverting the display. At higher, natural speeds, the percept was veridical with respect to the stimulus display. The authors interpreted these findings as evidence of the existence of biological motion channels tuned to higher, more natural walking speeds; channels that presumably dominate over a default assumption to perceive trajectories in depth [25]. This finding may be important in the current context. When the walker approaches angular orientations where image motion increases or decreases abruptly, changes in image motion may affect the structure/depth interpretation. For example, accelerating or decelerating movements of the hands and feet may trigger eye movements. These eye movements could then trigger perceptual alternations, with a limb moving in the opposite direction then becoming most salient, and triggering further eye movements (similar to the trapping that occurs with other ambiguous SFM, but different in the sense that it is not a ‘random’ dot that triggers eye movements). This could result in alternations congregating at systematic locations in the rotation cycle (and such patterns may be the reason observers find the ‘collapsing’ percept so frustrating).

We carried out control eye movement recordings with three observers. Each observer completed sixteen 180 s trials with the walker, eight trials with strict fixation and eight trials in which eye movements were encouraged. Eye movements were recorded using an Eyelink II (SR Research) eyetracker, which sampled binocular gaze positions at 250 Hz (pupil only). Much of our design and analysis was inspired by recent papers investigating eye movements and perceptual bistability [22]–[24], [26]. Details on experimental procedures and data analysis can be found in Materials & Methods. For this control experiment, we focused on observer gaze positions at the time of alternation to either of the two percepts (Mid-reversal i.e. 540–340 ms prior to button response) and in a post-reversal time window (Post-reversal i.e. 200 ms–0 ms prior to button response). We also analysed the distribution of saccadic eye movements relative to the rotation cycle of the walker.

The first result to note is that greater reversal rates were generally found when eye movements were allowed, although significant numbers of reversals still occurred when observers fixated (Figure 9B). Thus, while eye movements can facilitate reversal, it appears they may not be absolutely essential for reversal to occur [22] (although as we will see, eye movements appear to have been systematically triggered in the fixation condition). In Figure 9C, we present for each observer histograms that depict the distribution of saccadic eye movements relative to the stimulus' rotation cycle. To calculate these, we first collected all saccades made in a particular condition (8×3 min trials). We then calculated the proportion triggered during the presentation of individual frames of the rotation cycle, by noting the frame on screen during the starting sample of the saccade. Figure 9C presents the distributions for the fixation (blue) and eye movements encouraged (green) conditions, separated into fifteen equally sized bins (4 frames per bin). Also plotted is the distribution of image motion across the rotation cycle. For two observers in the fixation condition, the peak ‘trigger’ frame appears to occur at around frame 40 in the sequence, in a similar region to the image motion peak. Correlations between individual observers' saccade data and the image motion distribution, however, were somewhat weak. We also compared the distribution of motion contained in the higher salience dots (feet, hand/elbow) to the saccade distributions. Again, significant correlations were generally not apparent. A linear relationship between the distribution of motion energy in the stimulus and the probability of saccade occurrence is perhaps too simplistic a model. However, a significant positive correlation was found with observer TN for both fixation and eye movements encouraged conditions, when compared with the distribution of elbow/hand motion (Fixation: r = 0.527, p<0.05; Eye movements: r = 0.5592; p<0.05). This effect appeared to be driven by the motion of the ‘left’ hand dot (the dot that appears on the right of the screen at the beginning of Movie S1), with a very strong correlation between this dot's relative motion over the cycle and the distribution of TN's saccades over the cycle, for both conditions (Fixation: r = 0.706, p<0.005; Eye movements: r = 0.732, p<0.005). The motion of the opposite ‘right’ hand dot appeared not to have a comparable relationship to saccade occurrence for this observer (Fixation: r = 0.418, p = 0.12; Eye movements: r = 0.275, p = 0.32).

When we compare the distribution of saccades in the two conditions, a strong relationship is apparent for two observers across the fixation and eye movements encouraged conditions (AS: r = 0.728, p<0.005; SJ: r = −0.246, p = 0.378; TN: r = 0.6452, p<0.01; Combined: r = 0.714, p<0.005). We interpret this as an indication of the existence of an exogenous stimulus ‘trigger’ i.e. even when observers are asked to fixate, systematic eye movements appear to be triggered. Analyses of saccade direction also indicated that observers had preferences for saccades in particular directions, with horizontal and vertical saccades represented heavily. Finally, we also analysed the distribution of observers' button responses. The estimated reversal locations for each observer occurred in similar locations to their previous test sessions (Figure 6), with very similar histogram layouts in the fixation and eye movements encouraged conditions (AS: r = 0.788, p<0.0005; SJ: r = 0.944, p<10^−6^; TN: r = 0.922, p<10^−6^).

To examine whether fixation positions differed at the time of reversal towards each alternative percept, we also plotted the fixation positions separately for each type of transition (Switch to CW/Switch to CCW), focusing on the Mid-reversal (540 ms–340 ms prior to response) and Post-reversal (200 ms–0 ms prior to response) time windows. For each reversal, we found the median fixation positions during these two time windows. Figure 9D plots the average of these fixation positions across all reversals for each individual observer. Plots are separated by condition and transition type (although unlike in Experiment 3, both types of switch were monitored in each trial as normal). While systematic and facilitating eye movement patterns are clearly apparent in the eye movements encouraged condition, note the similarities in direction across observers, and the differences in direction for the two percept transition types. Switches to CW rotation led to obvious leftward eye movements for two observers, while switches to CCW led to rightward movements for all three observers. For each transition type, the mid-to-post reversal fixation differences were confirmed with t-tests on the horizontal position data. In the fixation condition, differences are much smaller between mid and post-reversal gaze positions. However, some significant differences still emerged for individual observers along specific directions [e.g. observer AS, switch to CW, vertical: t = 5.009, p<10^−3^ (means in visual angle: mid = −0.02, post = −0.098); observer TN, switch to CCW, horizontal: t = 2.5, p<0.05 (means in visual angle: mid = 0.046, post = 0.096)]. Significant mid-to-post reversal fixation differences were also found for other transition/direction pairings of observers SJ and TN in the fixation condition. Considered alongside the saccade distribution analysis, it appears that even in the fixation condition observers made some systematic eye movements. We conclude therefore that eye movement patterns play a significant role in shaping observers' experience with the stimulus, possibly playing a part in the formation of reversal location distributions.

## Discussion

Rotating structure from motion stimuli are part of a broad group of multistable stimuli employed by vision scientists to investigate the brain states and processes underlying our changing conscious awareness [4], [5]. By adapting a well-known stimulus type used in perceptual research, we add to this class of stimuli an ambiguous, rotating, biological figure. Current approaches to understanding the neural concomitants of bistable vision emphasise the distributed processes that are involved [4]. As biological motion is known to activate various visual cortical regions, including the EBA [10], pSTS [11], [12], and even higher-level visuo-motor regions [13], the potential for bistable biological motion to shed light on the hierarchical organisation of visual competition seems promising.

We first demonstrated the rapid and repeated perceptual switching that observers experience when a rotational component is added to a standard biomotion walker. On first viewing the stimulus, most naïve observers experienced relatively large numbers of perceptual reversals, easily comparable in frequency to reversals with a kinetic depth cylinder. The normalized phase durations and dominance times for CW/CCW were comparable for the two stimuli, giving a good first indication of the stimulus' bistable nature. In our second experiment, we investigated in more detail the temporal dynamics of reversal with biological motion, testing a small number of observers over four test repetitions on trials with standard walker and rotating walker stimuli. The phase distributions for individual observers showed good agreement across the various repetitions; the dynamics however differed in some ways for the two stimuli. For example, with standard biomotion, mean percept durations were consistently longer (in three out of four observers). This is an interesting finding, as it suggests that standard biomotion may be particularly useful in experimental situations where long percept durations are desired (e.g. fMRI [26]; see also [6]). Discounting observers' initial experience with the standard walker, during which the frontwards-facing percept usually dominates [17], over prolonged sessions the dominance towards the frontwards-facing percept is relatively small (58% of time on average). With the rotating walker, biases in two individual observer's data were more insidious and prolonged, lasting several sessions. It is difficult to understand the exact bases of these effects. Perhaps perceptual biases on initially viewing the stimulus, or the continual experience of set patterns of reversal (e.g. two rotations CCW, one rotation back) develop into longer patterns of dominance. The fact that no real dominance was found in two of the four observer's data suggests that these dominance patterns may be observer specific in nature (consider also TN's fifth and sixth repetitions, Figure 3B).

The reversal drift across trials was relatively minimal also, although slight differences were apparent for the two stimuli. A number of observers experienced drifts in the opposite direction for the two stimuli, with reversal rates with standard biomotion slowing over the trial for three observers. While the number of observers studied here was relatively small, one possible interpretation would be that the added rotational motion increases its ‘destabilizing’ effect over the course of a 120 s trial, perhaps due to processes of adaptation [27]–[29]. Detailed analysis of the phase distributions also confirmed that the maximum of the distributions for the rotating walker generally occurs earlier (in most cases at durations of 1 s or 2 s). In total, these results provide a compelling demonstration of the robustness of perceptual reversal with biological motion, and sit well alongside other recent findings with bistable phenomena [6], [7], [22].

We then carried out an experiment to investigate whether reversal hotspots exist with non-uniform rotating stimuli such as the walker. Re-analyses of the data from Experiments 1 and 2 had shown that some individual observers experienced peaks in the distribution of response locations relative to the stimulus' rotation cycle. While the data did not conclusively locate these ‘hotspots’, the fact that peaks also emerged for individual observers with a rotating cuboid stimulus, suggests that this perceptual toggling effect is not unique to the walker stimulus. We believe these patterns may be similar in nature to phenomena described previously [15], [22]. In our data, we found that several of the higher switchers in Experiment 1, and three of the four observers in Experiment 2, experienced reversals in very high proportion in particular quadrants of the rotation. Again, these locations are not necessarily the same for all observers. It may be the case that reaction time estimates are inaccurate by a greater amount for some observers, and that if a truly accurate measure of RT could be made, that a definite hotspot for reversal would be apparent. Alternatively, as indicated particularly by the cuboid, this perceptual toggling may be unique to particular observers and test repetitions, and possibly also to transition type (Switch to CW/Switch to CCW).

Finally, we must not forget that the fast rotational motion of the walker is likely to trigger eye movements. Asymmetries in the distribution of image motion could be a cause of distinct eye movement patterns that could trigger reversals more frequently at set points in the rotation. In control eye movement recordings, we found some evidence that saccades were triggered unequally across the rotation cycle, and that individual observers displayed preferences for saccades in particular directions. The distribution of saccades relative to the rotation cycle was highly correlated between the fixation and eye movements allowed conditions, supporting the notion of a strong exogenous trigger (e.g. image/dot motion). Gaze positions also differed in terms of mid-to-post reversal location, particularly when eye movements were allowed. A possible cause of these eye movement patterns may be found in specific features of the walker's motion. It may be that a particular limb movement consistently triggers a particular eye movement, regardless of which percept is currently perceived. If this is the explanation for reversal location peaks with the walker, it would explain why the reversal location peaks are in most cases based around a single position, and not multiple positions. For the rotating cuboid, in contrast, the peaks were based around two positions, on either side of the rotation cycle (note that the cuboid is symmetric at 180° intervals).

In conclusion, we have investigated in detail the temporal dynamics of perceptual reversal with biological motion. Over three experiments, we found that the temporal dynamics of alternation with biological motion are similar in many ways to reversals with other SFM stimuli. Observers experienced relatively stable patterns of percept reversal over sessions, for different types of biomotion display. In addition, characteristics of the stimulus (e.g. non-uniform structure), as well as characteristics of observers (e.g. eye movement patterns), may play interacting roles in the development of reversal patterns with biological motion.

## Materials and Methods

### Participants

A total of 21 observers (13 male) took part in these studies, including two authors. Except for author SJ and one other observer, participants in Experiment 1 (n = 17) were all naïve as to the nature of the stimuli. Participants in each of the remaining experiments had experience monitoring reversals with the walker, either in Experiment 1 or from practice sessions, and were aware of the nature of the stimuli, though naïve as to the purposes of the experiment. All observers could see depth in random-dot stereograms viewed through red-green glasses (one individual could not see depth in this screening test, and did not take part in further testing). Experimental sessions lasted approx. 60–90 mins with appropriate rest periods, and were carried out in accordance with local procedures and with the approval of UCD Research Ethics Committee. All participants gave written informed consent, and received a small hourly rate for participation.

### Experimental procedures

In Experiment 1, observers were trained to respond to changes in the direction of the walker and cylinder during separate 300 s practice trials, followed by 600 s test trials for each type of stimulus. Observers were not aware of the nature of the stimuli, but were instead instructed that the walker and cylinder would change direction (CW/CCW) at random intervals, sometimes with longer periods and other times with shorter periods in between consecutive reversals. Observers pressed one button on a response pad when the walker/cylinder switched to CW direction, and another for CCW. Observers were also instructed to press a third button if at any time a rotating three-dimensional form could not be discriminated from the stimulus dots, as pilot studies indicated approx. 10%–20% of naïve observers experienced short periods during a trial in which the walker percept disintegrates or ‘collapses’. We felt this third option might also allow observers to report additional interpretations that are possible with the kinetic-depth cylinder, although these were reported on few occasions. After all Experiment 1 testing, observers completed a post-test questionnaire relating to their impressions and experiences with the different stimuli. All observers also completed two control tasks–a 2-min trial with an unambiguous rotating cylinder (single convex surface, defined by a strong looming cue), during which rotation direction changed at pseudo-random intervals of between 2–12 s; and a 30-trial control task involving biomotion direction discrimination in noise. All observers performed close to ceiling in the controls (typically >90% accuracy). Two observers experienced the convex surface momentarily appearing concave, performing at 60%–70% accuracy; both performed at >90% accuracy for the biomotion control task, and their data were retained. All trials in Experiment 1 began with a 30 s pre-test period in which the stimuli were viewed continuously, so as to reduce the likelihood of observers becoming biased to one direction over the other at test onset.

In Experiment 2, observers completed trials with standard walker and rotating walker stimuli. For the standard walker stimulus, we presented a walker oriented at 30° left of frontwards, as though walking on a treadmill that faced beyond the observer's left shoulder. This figure can be perceived as either oriented towards (30°), or away from the observer (150°) via mirror reversal of the dots through the image plane (see Figure 1B, or [17]). We have found that people have difficulty experiencing this standard biomotion figure ‘switch’ for the first time, with the frontwards-facing percept invariably dominating at first. However, with practice it seems observers can experience quite stable and significant levels of reversal. Observers completed four repetitions of the experiment. Each repetition involved eight 120 s trials for each stimulus type, completed in random order and with appropriate rest periods between trials. For one observer (TN), two additional repetitions with the rotating walker were completed (six in total) in which the observer was encouraged to hold the less dominant percept.

Various control trials were also performed during the first session of Experiment 2, including the same control trials that observers completed in Experiment 1 (disambiguated cylinder, biomotion detection), as well as a number of control trials in which the standard and rotating stimuli were disambiguated. By presenting the walker with a small angular orientation difference between the left and right eyes' views, it is possible to create an unambiguous rotation direction for the rotating walker (and an unambiguous facing direction for the standard walker), as has previously been done with other SFM stimuli [29]. Using this method to present ‘catch’ trials, however, proved problematic, and in several trials observers genuinely report seeing reversals. With instruction, observers can detect the disambiguated direction quite well. It is possible that the strong form information in biomotion may over-rule the stereo information, particularly when observers are expecting reversals (i.e. in a ‘catch’ context). Each observer performed satisfactorily in the control cylinder and biomotion detection tasks.

In Experiment 3, observers completed three repetitions of twenty-four 1-min trials. Observers monitored one type of stimulus (walker or cuboid) and one type of transition (Switch to CW/Switch to CCW) in each block of six trials. Thus in total, 72 minutes of data was collected for each observer (2 stimuli×2 transition types×6 trials×3 repetitions). Some additional trials were completed with the rotating cuboid in the final session, or in a fourth session, as reversal rates were generally smaller for this stimulus. This was done so as to equate more closely the number of data points examined for each stimulus type.

### Stimuli

To create the rotating walker stimulus, we applied our own custom plotting functions over a publicly available motion capture recording of a walker on a treadmill [19], [20]. After re-sampling the coordinate data from 30 Hz to 60 Hz, we modified by hand the vertical position of a number of dots (see Coordinate Data), and then smoothed (2^nd^-order Butterworth, 10 Hz) and plotted the 3-D data in Matlab (The Mathworks, Inc.). We then rotated the camera viewing angle through 360° during a single step-cycle i.e. the viewing angle is rotated in Matlab in 6° steps for each of the 60 frames making up one revolution. Essentially, the walker remains ‘on the treadmill’, and the camera rotates around the walker (c.f. “The Matrix”, Warner Bros., 1999). By looping the resulting image sequence over multiple 1 s cycles, the walker appears to randomly alternate between walking in clockwise (CW) and counter-clockwise (CCW) directions (Movie S1). The walker consisted of 13 white dots (approx. 12 arcmin) and subtended approx. 7° of visual angle. The cylinder and cuboid stimuli were also created in Matlab, and for Experiments 1–3 stimulus presentation was controlled by a personal computer running Presentation software (www.neurobs.com). The rotating cylinder consisted of 120 leftward-moving and 120 rightward-moving white dots (2.4 arcmin), with a cycle period of 4 seconds, and wrap-around of dots when they reached the stimulus edge (i.e. dots that reached the right-hand edge were replaced again on the left). The velocity of each dot across the screen followed a sinusoidal profile, with speeds fastest in the middle and dropping away to zero at either edge; the two oppositely moving fields of dots appear to form opposite sides of a rotating cylinder. All stimuli were presented on a black background.

Stimuli in the first two experiments were presented monocularly using a head-mounted display (i-visor FX605, www.personaldisplay.com). Animation frames were synchronised to the display's refresh rate (60 Hz). No fixation point was used in Experiment 1; however, observers in Experiments 2 and 3 monitored a fixation dot throughout. The fixation dot in these trials and the eye movement recordings was always placed over the region of the torso (located above the elbow-line, approx. one-third of the distance to the shoulder-line). This is a fixation area that most observers find natural with biomotion. In experiments using the head-mounted display, participants viewed the stimuli through the left eye; the right half of the display remained blank. Participants indicated that stimuli remained clear and without conflict using this arrangement. The experimenter monitored and controlled stimulus presentation from a separate LCD display located away from the participant's station. Participants were supported with a chinrest during trials, and all testing was carried out in a darkened room. Stimuli in Experiment 3 were presented on a Sony Trinitron CRT; for the eyetracking control experiments a 21-inch Samsung SyncMaster 1100 MB was used.

### Eye movement recording

Gaze position was sampled at 250 Hz using an Eyelink II (SR-Research) eyetracker in conjunction with the Psychophysics Toolbox [30] and Eyelink Toolbox [31]. In our data analysis methods, we tried to follow as closely as possible the processing steps outlined in [22]–[24], [26], some of which are based on [32]. Participants completed two sets of eight 180 s trials (four per condition), with each set separated by approx. 1 hour rest. For the eye movement recordings, the walker was presented with an increased height of 11.2° visual angle, so that the walker's horizontal extent was magnified (approx. 4°–5°). Before each 180 s trial, the Eyelink system's nine-point calibration was run, and following this the system's drift correction was applied. To limit any variability introduced across different trials, blocks and set-ups, we also applied a further offline drift correction to the data, removing any components with a frequency below 0.1 Hz [26]. Blinks were detected as samples in which no pupil data was recorded. In addition, after calculating velocity (below), we searched in the 100 ms time windows before and after the missing pupil data and selected the earliest and latest samples with velocity greater than 12°/s. All samples inside and including these points were removed, as well as four additional samples either side, to limit the possibility of these samples being detected as saccades.

Gaze position data (in screen visual angles, see below) were median-filtered using a window width of nine samples. This process removes noise, while retaining sharp transitions. Velocities were calculated using a sliding window of five samples. To detect saccades, we searched for samples in which eye velocity exceeded 18°/s. Saccade start was set at the sample immediately preceding the rise above threshold, and saccade end was defined as the first sample after the velocity had reduced below 18°/s. From these candidate saccades, we retained only those that fulfilled both of the following criteria: (i) three samples or greater in duration (>12 ms) and (ii) binocular in nature. For binocular saccades, at least two samples overlapping in time were required. For the analyses, we only studied saccades with an amplitude of 8° or less (the vast majority lay in the region 0.5°–3° amplitude), and saccades that remained within a region measuring 6° across by 12° high. Our saccade detection routine identified a small number (<15) of high amplitude/low velocity candidate saccades in observer TN's data, which we removed by hand. Saccade direction was measured as the angle between start and end position samples, and saccades were related to the stimulus rotation cycle by noting which frame was on screen during the starting sample of the saccade.

Despite the Eyelink system's high resolution, our use of gaze position data (rather than eye rotation angles) may introduce slight inaccuracies in the velocity calculation, which may not be truly representative of eye rotation velocity i.e. our measure reflects more accurately the speed of gaze displacement across the screen. To confirm that our saccade detection still performed satisfactorily, we examined all of the saccade data extracted and verified that the distributions for each observer conform roughly to the ‘main sequence’ [32] (see insets in Fig. 9B).

### Stimulus Coordinate Data

Although the raw 3-D coordinate data [19], [20] depicting treadmill walking is symmetric about the vertical axis (i.e. the left half of the body is identical to the right, but 180° out of phase), when the camera is gradually rotated around the walker in Matlab, asymmetries are apparent in the vertical position of different dot pairs during the rotation cycle e.g. one elbow dot appears higher than the other when they cross paths in the image. We felt this asymmetry may have provided an unwanted perspective cue, particularly for the elbow dot pair, whose characteristic opponent motions are known to be particularly salient features of biological motion [13]. We made minimal corrections to the vertical position of the elbow dot pair before smoothing and plotting. The coordinate data used to create the rotating walker can be obtained from the present authors on request. Details of the raw treadmill recording can be obtained from references [19] and [20].

### Data analysis

All analyses and data summaries were carried out using R routines (www.r-project.org), except the polar plot analyses mapping participants' responses to stimulus orientation, and the eye movement data, which were analysed using Matlab functions (The Mathworks, Inc.).

## Supporting Information

Movie S1 An ambiguous, rotating, biological figure
(0.02 MB MOV)

## References

[1] Blake R, Fox R (1974). Adaptation to invisible gratings and the site of binocular rivalry suppression.. Nature.

[2] Tong F, Nakayama K, Vaughan JT, Kanwisher N (1998). Binocular rivalry and visual awareness in human extrastriate cortex.. Neuron.

[3] Dodd JV, Krug K, Cumming BG, Parker AJ (2001). Perceptually bistable three-dimensional figures evoke high choice probabilities in cortical area MT.. J Neurosci.

[4] Blake R, Logothetis NK (2002). Visual competition.. Nat Rev Neurosci.

[5] Leopold DA, Logothetis NK (1999). Multistable phenomena: changing views in perception.. Trends Cogn Sci.

[6] Van Ee R (2005). Dynamics of perceptual bi-stability for stereoscopic slant rivalry and a comparison with grating, house-face, and Necker cube rivalry.. Vis Res.

[7] Suzuki S, Grabowecky M (2007). Long-term speeding in perceptual switches mediated by attention-dependent plasticity in cortical visual processing.. Neuron.

[8] Carter O, Cavanagh P (2007). Onset rivalry: brief presentation isolates an early independent phase of perceptual competition.. PLoS ONE.

[9] Johansson G (1973). Visual perception of biological motion and a model for its analysis.. Percept Psychophys.

[10] Downing PE, Jiang Y, Shuman M, Kanwisher N (2001). A cortical area selective for visual processing of the human body.. Science.

[11] Bonda E, Petrides M, Ostry D, Evans A (1996). Specific involvement of human parietal systems and the amygdala in the perception of biological motion.. J Neurosci.

[12] Grossman E, Donnelly M, Price R, Pickens D, Morgan V (2000). Brain areas involved in perception of biological motion.. J Cogn Neurosci.

[13] Giese MA, Poggio T (2003). Neural mechanisms for the recognition of biological movements.. Nat Rev Neurosci.

[14] Kourtzi Z, Krekelberg B, Van Wezel RJA (2008). Linking form and motion in the primate brain.. Trends Cogn Sci.

[15] Wallach H, O'Connell DN (1953). The kinetic depth effect.. J Exp Psych.

[16] Necker LA (1832). Observations on some remarkable optical phenomena seen in Switzerland; and on an optical phenomenon which occurs on viewing a figure of a crystal or geometrical solid.. Lond Edinburgh Phil Magazine J Sci.

[17] Vanrie J, Dekeyser M, Verfaillie K (2004). Bistability and biasing effects in the perception of ambiguous point-light walkers.. Perception.

[18] Watson TL, Pearson J, Clifford CWG (2004). Perceptual grouping of biological motion promotes binocular rivalry.. Curr Biol.

[19] Vanrie J, Verfaillie K (2004). Perception of biological motion: A stimulus set of human point-light actions.. Behav Res Meth Instr & Comp.

[20] Vanrie J, Verfaillie K (2004). Vanrie-BRMIC-2004.zip.. http://www.psychonomic.org/archive/.

[21] Hupe JM, Rubin N (2003). The dynamics of bi-stable alternation in ambiguous motion displays: a fresh look at plaids.. Vis Res.

[22] Brouwer GJ, Van Ee R (2006). Endogenous influences on perceptual bistability depend on exogenous stimulus characteristics.. Vis Res.

[23] Van Dam LCJ, Van Ee R (2006). Retinal image shifts, but not eye movements per se, cause alternations in awareness during binocular rivalry.. J Vis.

[24] Van Dam LCJ, Van Ee R (2006). The role of saccades in exerting voluntary control in perceptual and binocular rivalry.. Vis Res.

[25] Beintema JA, Oleksiak A, Van Wezel RJA (2006). The influence of biological motion perception on structure-from-motion interpretations at different speeds.. J Vis.

[26] Brouwer GJ, Van Ee R (2007). Visual cortex allows prediction of perceptual states during ambiguous structure-from-motion.. J Neurosci.

[27] Nawrot M, Blake R (1991). A neural network model of kinetic depth.. Vis Neurosci.

[28] Fang F, He S (2004). Stabilized structure from motion without disparity induces disparity adaptation.. Curr Biol.

[29] Nawrot M, Blake R (1991). The interplay between stereopsis and structure from motion.. Percept Psychophys.

[30] Brainard DH (1997). The Psychophysics Toolbox.. Spat Vis.

[31] Cornelissen FW, Peters EM, Palmer J (2002). The Eyelink Toolbox: Eye tracking with Matlab and the Psychophysics Toolbox.. Behav Res Meth Instr & Comp.

[32] Engbert R, Kliegl R (2003). Microsaccades uncover the orientation of covert attention.. Vis Res.

[33] Brascamp JW, Van Ee R, Pestman WR, Van den Berg AV (2005). Distributions of alternation rates in various forms of bistable perception.. J Vis.

[34] Mamassian P, Landy MS (1998). Observer biases in the 3D interpretation of line drawings.. Vis Res.

